# Ketamine treatment alleviates suicide ideation in high-risk populations: a systematic review and meta-analysis

**DOI:** 10.1017/S2045796025100371

**Published:** 2026-01-12

**Authors:** Wen Tang, Wei-Wei Jiang, Wen-Qian Que, Wan-Qing Zhang, Hong-Lin Chen, Li-Juan Zhou

**Affiliations:** 1 Nursing Department, The Affiliated Taizhou People’s Hospital of Nanjing Medical University, Taizhou, China; 2 School of Nursing and Rehabilitation, Nantong University, Nantong, China; 3 Nursing department, Affiliated Qidong Hospital of Nantong University, Nantong, China; 4 Nursing department, Qidong People’s Hospital, Nantong, China; 5 Qidong Liver Cancer Institute, Nantong, China; 6 Nursing Department, Xuzhou Central Hospital, Xuzhou, China

**Keywords:** ketamine, meta-analysis, moderator, suicide ideation, treatment-resistant depression

## Abstract

**Aims:**

To synthesize the available experimental study evidence to estimate the effects of ketamine on suicide ideation (SI) in high-risk individuals.

**Methods:**

We conducted a systematic review and meta-analysis following the Preferred Reporting Items for Systematic Reviews and Meta-Analyses (PRISMA) guidelines. Double-blind randomized controlled trials and open-label studies investigating the safety and effectiveness of ketamine on SI published up to October 2025 were identified. Data were pooled using random-effects meta-analysis. The main outcome was standardized mean difference on SI in high-risk individuals. Secondary outcomes were the percentage of adverse events and the moderator effects.

**Results:**

We identified 21 studies with a total of 927 participants meeting our inclusion criteria. The pooled effect size for the reduction of SI after ketamine treatment was significant and clinically meaningful (large effect size of −1.40, 95% confidence interval: −2.15 to −0.66, *P* < 0.001, low–quality evidence). Dissociation (38.8%, *P* = 0.014), nausea (31.6%, *P* < 0.001), dizziness (24.7%, *P* = 0.003), headache (22.0%, *P* = 0.011) and anxiety (15.8%, *P* < 0.001) were the frequently reported adverse events. Moderator analyses indicated that the effect was higher in younger individuals and those with severe SI.

**Conclusions:**

Our findings highlight the effectiveness of ketamine in reducing SI in high-risk individuals, especially younger individuals and those with severe ideation. Nonetheless, additional research is required to better understand optimal dosing regimens and the potential long-term effects of ketamine treatment.

## Introduction

Suicide is a growing public health concern with ramifications for families, friends, coworkers, communities and society. The World Health Organization reports that globally, more than 700,000 people die due to suicide every year (World Health Organization, 2023). Depression, anxiety, post-traumatic stress disorder (PTSD) are well-established risk factors for suicide and the emergence of suicide ideation (SI) (Heinrich *et al.*, 2022; Turecki *et al.*, 2019; Urban *et al.*, 2013). The pathophysiology of depression and SI is complex, involving dysregulation of neurotrophic factors like brain-derived neurotrophic factor (BDNF), neuroinflammation and oxidative stress (Guan *et al.*, 2021). Despite the availability of various treatment options, including medications and psychotherapies, SI remains difficult to treat in some patients, particularly those who are at high risk for suicide (Zalsman *et al.*, 2016).

Ketamine, a noncompetitive N-methyl-D-aspartate receptor antagonist, has been utilized as an anaesthetic since the 1960s (Zhang *et al.*, 2023). Besides its anaesthetic properties, ketamine has also been proven to have antidepressant effects (Bartoli *et al.*, 2017; Marcantoni *et al.*, 2020; Yin *et al.*, 2024). The antidepressant effects of ketamine are believed to be due to its ability to enhance the production of brain-derived neurotrophic factor, an essential protein for neuronal growth and survival. This aligns with a broader understanding that the hippocampal CRTC1-CREB-BDNF pathway is critical for antidepressant efficacy (Liu *et al.*, 2020). Ketamine has been used off-label for treating depression, anxiety, PTSD and other psychiatric disorders, and has shown quick and prolonged antidepressant effects in some patients (Singh *et al.*, 2016).

The rapid and sustained antidepressant effects of ketamine render it a promising candidate for treating SI. Studies have established that ketamine can promptly diminish SI in patients suffering from depression, bipolar disorder, PTSD and other psychiatric disorders. Moreover, ketamine’s anti-suicidal effects seem to be unrelated to its antidepressant effects, indicating that ketamine may employ distinct mechanisms of action to mitigate SI (Ballard *et al.*, 2014; Grunebaum *et al.*, 2017).

Despite promising results from individual studies, a consensus on the effectiveness and safety of ketamine in reducing SI has not been reached. Therefore, this study aims to conduct a systematic review and meta-analysis to comprehensively evaluate the existing literature on ketamine treatment for SI in high-risk suicide groups, and to determine the overall efficacy and safety of ketamine in reducing SI in this population. The study will also explore potential moderators of the effect of ketamine on SI, such as dose, frequency of administration, duration of treatment and baseline clinical characteristics. The findings of this review will provide valuable insights into the potential role of ketamine in managing suicide risk, and will inform clinical practice and future research in this area.

## Methods

This study complies with PRISMA 2020 recommendations (Page *et al.*, 2021). The protocol was registered in the International Prospective Register of Systematic Reviews database (ID: CRD42023415655).

### Eligibility criteria

We included studies if they met the following criteria:

1. Type of studies: Experimental and observational studies, including randomized trials, open-label, cohort and case-control studies were considered for inclusion. The search was limited to studies published in English.
2. Type of participants: Individuals identified as high-risk for SI, as determined by various suicide assessment scales, without restrictions on sex, age, or race.
3. Type of interventions: Treatment with ketamine intervention, which can be performed using any route of administration. For example, intravenous, intramuscular, oral, or inhalation administration.
4. Type of control: The comparison intervention was routine treatment, no treatment or placebo (i.e., saline, midazolam and electroconvulsive therapy).
5. Type of outcomes: Studies that reported raw data or statistics related to SI as measured by any type of scale (e.g., SPS, ASIQ, CSSRS, MADRS-SI, BSI, QIDS-SI, etc.) were considered for inclusion.

### Exclusion criteria

We excluded studies that met any of the following criteria:

1. Type of studies: Abstracts of conference proceedings, study protocols, media news, commentaries, reviews, or case reports were excluded.
2. Type of participants: Studies that did not report SI data were excluded.
3. Type of interventions: Studies with unclearly reported interventions were excluded.
4. Type of control: Studies with incomplete control group data were excluded.
5. Type of outcomes: Studies with incomplete baseline data of participants were excluded.

### Primary and secondary outcomes

The pre-defined primary outcome was the standardized mean difference (SMD) in SI scores measured by validated scales (e.g., CSSRS, MADRS-SI) between the ketamine and control groups at post-treatment.

Secondary outcomes included: (1) Prevalence of treatment-emergent adverse events (e.g., dissociation, nausea); (2) Moderator effects of age, administration treatment, frequency of medication, control, assessment tool, health status, administration route and study design.

### Search strategy

In this systematic review and meta-analysis, two independent reviewers conducted a comprehensive search for studies evaluating the efficacy of ketamine in reducing SI. The search included various types of publications, including peer-reviewed publications and preprints, and was conducted across multiple databases (PubMed, Web of Science, medRxiv, EMBASE) using controlled vocabulary keywords such as ‘ketamine’, ‘racemic ketamine’, ‘esketamine’, ‘suicide ideation’ and ‘suicidal ideation’ (complete search syntax in Table S1).

Our inclusion criteria were restricted to studies published between January 2003 and October 2025 to capture the most contemporary evidence reflecting the paradigm shift in ketamine research following its Food and Drug Administration (FDA)-approval trajectory (U.S. Food and Drug Administration, 2019), evolving diagnostic criteria in DSM-IV-TR (Association, 2000), and standardized measurement tools in suicidology research (e.g., CSSRS 2003). When encountering multiple reports from the same cohort, we prioritized the largest/most complete dataset to prevent patient duplication. To ensure comprehensiveness, we conducted a thorough search for references cited in prior systematic reviews.

### Study selection and data extraction

To ensure accuracy, we removed any duplicated studies, and then had two independent reviewers select the remaining studies based on the eligibility criteria. They examined the titles, abstracts, or full texts to make their selections. For each chosen study, we extracted sample size, participant characteristics, mean scores of SI, and the corresponding standard deviation (SD) to calculate the effect size. Safety outcomes were evaluated by the prevalence of adverse events. If data were unclear, we contacted the authors for clarification. If no response was received, the study was excluded. We combined data from multi-arm trials into a single comparison group. In cases of disagreement regarding study selection or data extraction, we resolved the issue through discussion or by consulting a third reviewer.

In order to investigate the correlation between ketamine treatment and SI, we collected additional information regarding trial characteristics and participants. The characteristics included the age, sex and health status of the participants, as well as the frequency, route, reaction time of administration, the proportion of adverse events, and whether the assessment was blinded to the intervention. The availability of SI measurement tools was also taken into account. We did not collect data regarding race and ethnicity as this was outside the study scope.

### Assessment of bias and methodological quality

Two reviewers independently assessed the methodological quality of studies using the National Institutes of Health Quality Assessment Tools (National Health Lung and Blood Institute, 2021), selected for their ability to evaluate diverse study designs included in our analysis. The evaluation items in each tool included study participants, sample size justification, treatment allocation, measures of exposure and other biases. Each item was evaluated with possible answers being yes, no, or other. Disagreements were resolved either through discussion or with the assistance of a third reviewer. After the evaluation, we assigned each study a quality rating of ‘good’, ‘fair’, or ‘poor’. Regardless of the quality rating, all studies were treated equally in the primary analysis. In addition, we employed a funnel plot and used Egger and Begg’s test to assess the publication bias (or small-study effects).

Two independent reviewers assessed the quality of evidence in this review according to the Grading of Recommendations Assessment, Development and Evaluation approach (Atkins *et al.*, 2004). The quality of evidence was rated as high, moderate, low, or very low based on several factors, such as study limitations, inconsistency, indirectness, imprecision and other relevant factors. In cases of disagreement, we resolved the issue through discussion or by consulting a third reviewer.

### Data analysis

Using R and R Studio (Version 4.2.0), we conducted the meta-analysis by employing a random-effects model to calculate pooled estimates (SMD) and the corresponding 95% confidence interval (CI) to account for variations in participants and assessment instruments used for SI across studies. Adverse events were quantified as event prevalence with 95% CI. For each comparison, we estimated Cohen’s d using post-treatment or follow-up means, SDs and sample sizes, where a negative effect size suggested that ketamine therapy was more effective than the control condition or baseline. The safety of ketamine treatment was analysed using random-effects pairwise meta-analysis and was reported as prevalence and 95% CI. To assess between-study heterogeneity, we used the Cochran Q test. The percentage of variability that may be attributed to between-study heterogeneity rather than sampling error was calculated using the Higgins *I*^2^ statistic, with *I*^2^ values of 25%, 50% and 75%, respectively, denoting low, moderate and high levels of heterogeneity. Our meta-analysis accounted for variations across studies, using appropriate statistical tests to ensure accurate and reliable results (Higgins *et al.*, 2003).

In order to evaluate potential publication bias, we employed two methods: a visual examination of the symmetry of the funnel plot and an Egger regression test. A *P*-value below 0.05 in the Egger regression test was indicative of significant publication bias. Moreover, we conducted sensitivity analyses by re-computing the effect size after eliminating possible outliers (Egger *et al.*, 1997). We classified studies as outliers if their 95% CI of effect size did not overlap with the 95% CI of the overall effect size (Viechtbauer and Cheung, 2010).

To assess the impact of potential moderators on the overall effect size and to explore any possible heterogeneity, we conducted meta-regressions. We selected three main moderators a priori: age of participants, evaluation time points and duration of medications. These moderators were chosen based on their possible associations with SI, ketamine treatment, or both and previous meta-analyses on ketamine treatment for SI in adults showed significant associations with similar moderators. To examine the impact of four primary moderators, we utilized multiple linear regression analyses with a mixed-effects model that employed maximum likelihood estimation. Furthermore, we conducted univariable analyses to explore whether participant and trial characteristics could moderate the overall effect. We considered a two-sided *P*-value of less than 0.05 to be significant. For detailed information on all moderators and their codes, please refer to eTable 1 in the Supplement.

## Results

The initial database search yielded 1,669 unique records. After screening titles and abstracts, we assessed 104 full texts, and ultimately included 21 studies in the meta-analysis.

### Study characteristics

The main characteristics of the included studies are summarized in Table 1. Of the 21 studies included, 13 were randomized double-blind controlled trials (RCTs), 8 were open-label studies, and included 927 participants (509 [54.9%] female; 418 [45.1%] male). The mean (SD) age of participants at baseline was 38.1 (13.2) years. Out of the 21 included studies, 16 studies enrolled participants with a psychiatric disorder, including depression, depressive disorder and treatment-resistant depression (TRD).

**Table 1.** Characteristics of included studies

Study (author, year)	Country	Design	Sample size/female	Diagnosis at recruitment	Age (y, mean ± SD)	Evaluation indicators	Administration	Treatment	Key finding
Ahmed GK, 2023 (Ahmed *et al.*, 2023)	Egypt	Double-blind RCT	36/15	MDD	36.36 ± 13.54	SPS	Intervention: Ket (0.5 mg/kg, 40 min, i.v.) Control: Placebo (saline)	Repeated (2 weeks, once weekly)	①Ketamine infusions in treatment-resistant depression reduce SI.
Gaither R, 2022 (Gaither *et al.*, 2022)	USA	Open–label study	14-Jul	Active SI	36.1 ± 13.1	CSSRS	Ket (0.5 mg/kg, 40 min, i.v.)	Single	①Ketamine was shown to be well-tolerated and exhibit short-term effectiveness. ②The improvements in mood and reductions in suicidality were observed to persist at the 6-hour mark.
Hochschild A, 2022 (Hochschild *et al.*, 2022)	USA	Double-blind RCT	80/48	MDD	39.55 ± 13.1	BSI	Intervention: Ket (0.5 mg/kg, 40 min, i.v.) Control: Midazolam (0.02 mg/kg, i.v.)	Single	①Ketamine has rapid effects on SI.
Peters EM, 2022 (Peters *et al.*, 2022)	Canada	Open-label study	30/21	TRD	31.34 ± 10.25	MADRS-SI	Ket (Protocol 1, 50 mg/day, four consecutive weekdays, IN; Protocol 2, 50 mg + 75 mg every second day, IN)	Repeated (Protocol 1: 50 mg/day, consecutive 4 days; Protocol 2: 50 mg (Day 1) followed by 75 mg every second day (Days: 2, 4, 6)	①Compounded intranasal racemic ketamine was safe and effective in the treatment of SI.
Shivanekar S, 2022 (Shivanekar *et al.*, 2022)	USA	Open-label study	32/16	Active SI	34.81 ± 16.01	CSSRS ASIQ BSI	Intervention: Ket (0.5 mg/kg, 40 min, i.v.) Control: Placebo (saline)	Single	①The rapid reduction of SI was observed 2−24 hours after a single infusion of ketamine in patients with high SI. ②These improvements were consistently sustained for up to 6 months after infusion.
Zhou Y, 2022 (Zhou *et al.*, 2022)	China	Open-label study	86/47	MDD or bipolar depressive disorder	33.5 ± 11.2	BSI	Ket (0.5 mg/kg, 40 min, i.v.)	Repeated (2 weeks, thrice weekly)	①Ketamine has anti-suicide effects.
Feeney A, 2021 (Feeney *et al.*, 2021)	USA	Double-blind RCT	40/16	TRD	45.75 ± 12.32	MADRS-SI	Intervention: Ket (0.1 mg/kg, 0.5 mg/kg or 1.0 mg/kg, i.v.) Control: Midazolam (0.045 mg/kg, i.v.)	Single	①A single infusion of IV ketamine may reduce SI out to 30 days post-infusion, but early anti-suicidal effects appear to diminish rapidly.
Keilp JG, 2021 (Keilp *et al.*, 2021)	USA	Double-blind RCT	78/47	MDD	38.4 ± 12.92	BSI	Intervention: Ket (0.5 mg/kg, 40 min, i.v.) Control: Midazolam (0.02 mg/kg, i.v.)	Single	①Ketamine produced rapid improvement in SI and mood in comparison to midazolam.
Pathak U, 2021 (Pathak *et al.*, 2021)	India	Double-blind RCT	60/34	Depression	31.68 ± 12.41	MSSI	Intervention: Ket (0.5 mg/kg, 40 min, i.v.) Control: Placebo (saline)	Single	①There were significant reductions in MSSI score within 6 h of the first dose in the ketamine group as compared to the normal saline group.
Kheirabadi D, 2020 (Kheirabadi *et al.*, 2020)	Iran	Double-blind RCT	39/19	MDD	40.72 ± 11.08	BSI	Group 1: Ket (1 mg/kg, oral) Group 2: 6–9 sessions of ECT Group 3: Ket (0.5 mg/kg, IM)	Repeated (Oral: 3 weeks, 6–9 sessions; IM: 3 weeks, 6–9 injections)	①Oral and IM ketamine probably have equal anti-suicidal effect.
Phillips JL, 2020 (Phillips *et al.*, 2020)	Canada	Open-label study	37/19	TRD	39.5 ± 11.7	MADRS-SI	Intervention: Ket (0.5 mg/kg, 40 min, i.v.) Control: Midazolam (30 µg/kg, i.v.)	Single	①A larger reduction in SI was elicited by a single ketamine infusion, with maximal effects measured at 7 days post-infusion.
								Repeated (2 weeks, thrice weekly)	①Repeated ketamine infusions given thrice weekly resulted in a progressive reduction in MADRS-SI scores.
Zhou Y, 2020 (Zhou *et al.*, 2020)	China	Open-label study	73/35	Unipolar or bipolar depression	33.75 ± 11.65	BSI	Ket (0.5 mg/kg, 40 min, i.v.)	Repeated (2 weeks, thrice weekly)	①Repeated ketamine treatment could be considered an eligible predictor for acute- and short-term response for treating depression with SI.
Ionescu DF, 2019 (Ionescu *et al.*, 2019)	USA	Double-blind RCT	26-Oct	TRD	45.4 ± 12.4	CSSRS	Intervention: Ket (0.5 mg/kg, 45 min, i.v.) Control: Placebo (saline)	Repeated (3 weeks, twice weekly)	①Ketamine did produce better effects on SI than placebo but not significantly.
Canuso CM, 2018 (Canuso *et al.*, 2018)	USA	Double-blind RCT	66/43	MDD	35.8 ± 13.03	BSI	Intervention: Ket (84 mg, IN) Control: Placebo (saline)	Repeated (4 weeks, twice weekly)	①Intranasal esketamine may result in significantly rapid improvement in SI.
Sinyor M, 2018 (Sinyor *et al.*, 2018)	Canada	Double-blind RCT	09-May	MDD	32.78 ± 7.48	BSI MADRS-SI	Intervention: Ket (0.5 mg/kg, 40 min, i.v.) Control: Midazolam (0.045 mg/kg, i.v.)	Repeated (2 weeks, thrice weekly)	①It showed a greater than two-fold reduction in SI following ketamine infusions.
Fan W, 2017 (Fan *et al.*, 2017)	China	Double-blind RCT	37/25	Cancer	45.78 ± 14.37	BSI MADRS-SI	Intervention: Ket (0.5 mg/kg, 40 min, i.v.) Control: Midazolam (0.05 mg/kg, i.v.)	Single	①Both BSI and MADRS-SI scores were significantly lower in the ketamine group compared to the midazolam group on day 1 and day 3.
Hu YD, 2016 (Hu *et al.*, 2016)	China	Double-blind RCT	27/17	MDD	39.0 ± 12.6	QIDS-SI	Intervention: Ket (0.5 mg/kg, 40 min, i.v.) Control: Placebo (saline)	Single	①By 4 weeks, more ketamine-treated patients had responded and remitted than placebo-treated patients, and the response and remission time were significantly shorter. ②Ketamine was found to be associated with significantly lower QIDS-SI scores from 2 to 72 hours compared to placebo.
Murrough JW, 2015 (Murrough *et al.*, 2015)	USA	Double-blind RCT	24/16	MDD, bipolar disorder, PTSD	42.4 ± 13.3	MADRS-SI	Intervention: Ket (0.5 mg/kg, 40 min, i.v.) Control: Midazolam (0.045 mg/kg, i.v.)	Single	①At 48 hours following intervention, a significant effect of ketamine treatment on BSI score was observed. ②The ketamine group had significantly lower MADRS-SI scores compared to the midazolam group 24 hours after treatment. The effect was not significant at 48 h, 72 h or 7 days.
Price RB, 2014 (Price *et al.*, 2014)	USA	RCT	57/30	TRD	46.83 ± 11.36	BSI MADRS-SI QIDS-SI	Intervention: Ket (0.5 mg/kg, 40 min, i.v.) Control: Midazolam (0.045 mg/kg, i.v.)	Single	①Intravenous administration of ketamine leads to rapid reductions in suicidal ideation, surpassing the effects of placebo.
Kashani P, 2014 (Kashani *et al.*, 2014)	Iran	Open-label study	49/25	Active SI	32 ± 10.8	BSI	Ket (0.2 mg/kg, over one minute, i.v.)	Single	①BSI scores significantly dropped after ketamine injection.
Thakurta RG, 2012 (Thakurta *et al.*, 2012)	India	Open-label study	27/14	MDD	49.44 ± 8.10	BSI HDRS-SI	Ket (0.5 mg/kg, 40 min, i.v.)	Single	①The ketamine infusion demonstrated effectiveness in reducing both BSI and HDRS-SI scores, with significant changes observed at each time point from minute 40 to 230.

ASIQ, Adult Suicidal Ideation Questionnaire; BSI, Beck Scale for Suicidal Ideation; CSSRS, Columbia-Suicide Severity Rating Scale; ECT, emission computed tomography; IM, intramuscular; IN, intranasal; i.v., intravenous; Ket, ketamine; MADRS-SI, Montgomery-Asberg Depression Rating Scale – Suicidal Ideation; MDD, major depressive disorder; MSSI, Modified Scale for Suicidal Ideation; PTSD, post-traumatic stress disorder; QIDS-SI, Quick Inventory of Depressive Symptomatology – Suicidal Ideation item; SI, suicide ideation; SPS, Suicide Probability Scale; TRD, treatment-resistant depression.

The frequency of the ketamine treatment ranged from one to four per week, with thrice weekly being the most commonly used frequency in repeated treatment (four studies [Phillips *et al.*, 2020; Sinyor *et al.*, 2018; Zhou *et al.*, 2020, 2022]). Two studies evaluated intranasal administration of ketamine (Canuso *et al.*, 2018; Peters *et al.*, 2022), 1 study evaluated intramuscular injection (Kheirabadi *et al.*, 2020), 1 study evaluated oral administration (Kheirabadi *et al.*, 2020), and 18 studies evaluated the efficacy of intravenous infusion (Ahmed *et al.*, 2023; Fan *et al.*, 2017; Feeney *et al.*, 2021; Gaither *et al.*, 2022; Hochschild *et al.*, 2022; Hu *et al.*, 2016; Ionescu *et al.*, 2019; Kashani *et al.*, 2014; Keilp *et al.*, 2021; Murrough *et al.*, 2015; Pathak *et al.*, 2021; Phillips *et al.*, 2020; Price *et al.*, 2014; Shivanekar *et al.*, 2022; Sinyor *et al.*, 2018; Thakurta *et al.*, 2012; Zhou *et al.*, 2020, 2022). More information regarding the ketamine treatment of the included studies is available in eTable 2 in the Supplement.

### Mean effect size, heterogeneity and bias

**Figure 1.**
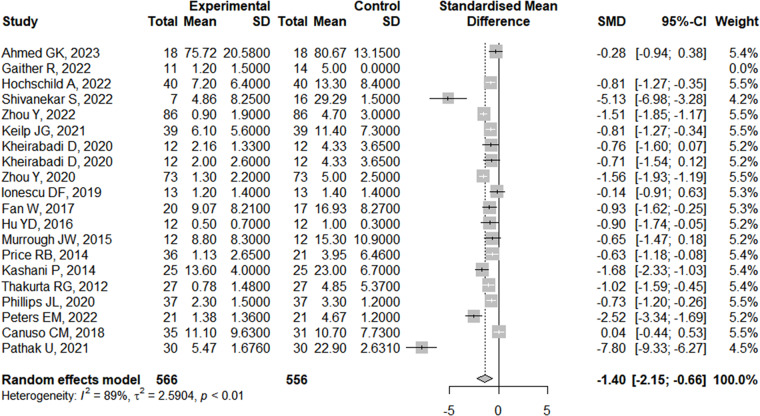
Forest plot of SMD in patients allocated to the ketamine group and the control group.

Figure 1 provides a visual representation of the distribution of the effect sizes of the included studies. It can be seen that none of the effect sizes is greater than 0, indicating larger reductions in SI with ketamine treatment compared to control conditions or baseline. The ketamine treatment had a mean effect size of −1.40 (SMD, 95% CI: −2.15 to −0.66, *P* < 0.001, low–quality evidence).

Only six studies assessed the effects of ketamine treatment on SI at follow-up (Feeney *et al.*, 2021; Hu *et al.*, 2016; Shivanekar *et al.*, 2022; Sinyor *et al.*, 2018; Zhou *et al.*, 2020, 2022). The median follow-up period post-administration was 1 month (range, 2 weeks – 6 months). Efficacy in follow-up at Week 2 was −2.05 (SMD, 95% CI: −3.16 to −0.94, *P* < 0.001, low–quality evidence), indicating that ketamine was effective for alleviating SI at Week 2. No association of ketamine treatment with SI was detected in follow-up at Month 1 and Month 6 (Month 1: Cohen’s *d* = −1.417, 95% CI: −3.03 to 0.20, *P* = 0.086, very low–quality evidence; Month 6: Cohen’s *d* = −1.73, 95% CI: −5.60 to 2.14, *P* = 0.381, very low–quality evidence) (Figure S1). Overall, the differences were no longer statistically significant after 2 weeks (*P* > 0.05)

Only six studies assessed the effects of ketamine treatment on SI at follow-up (Feeney *et al.*, 2021; Hu *et al.*, 2016; Shivanekar *et al.*, 2022; Sinyor *et al.*, 2018; Zhou *et al.*, 2020, 2022). The median follow-up period post-administration was 1 month (range, 2 weeks – 6 months). Efficacy in follow-up at Week 2 was −2.05 (SMD, 95% CI: −3.16 to −0.94, *P* < 0.001, low–quality evidence), indicating that ketamine was effective for alleviating SI at Week 2. No association of ketamine treatment with SI was detected in follow-up at Month 1 and Month 6 (Month 1: Cohen’s *d* = −1.417, 95% CI: −3.03 to 0.20, *P* = 0.086, very low–quality evidence; Month 6: Cohen’s *d* = −1.73, 95% CI: −5.60 to 2.14, *P* = 0.381, very low–quality evidence) (Figure S1). Overall, the differences were no longer statistically significant after 2 weeks (*P* > 0.05)

Only six studies assessed the effects of ketamine treatment on SI at follow-up (Feeney *et al.*, 2021; Hu *et al.*, 2016; Shivanekar *et al.*, 2022; Sinyor *et al.*, 2018; Zhou *et al.*, 2020, 2022). The median follow-up period post-administration was 1 month (range, 2 weeks – 6 months). Efficacy in follow-up at Week 2 was −2.05 (SMD, 95% CI: −3.16 to −0.94, *P* < 0.001, low–quality evidence), indicating that ketamine was effective for alleviating SI at Week 2. No association of ketamine treatment with SI was detected in follow-up at Month 1 and Month 6 (Month 1: Cohen’s *d* = −1.417, 95% CI: −3.03 to 0.20, *P* = 0.086, very low–quality evidence; Month 6: Cohen’s *d* = −1.73, 95% CI: −5.60 to 2.14, *P* = 0.381, very low–quality evidence) (Figure S1). Overall, the differences were no longer statistically significant after 2 weeks (*P* > 0.05)

**Figure 2.**
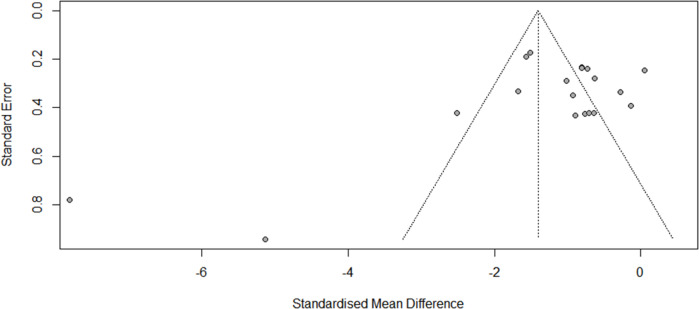
Funnel plots of the publication bias.

There was evidence of high heterogeneity at post-administration (*I*^2^ = 89, *P* < 0.001) and at follow-up (Week 2: *Q* = 41.66; df = 3, *I*^2^ = 92.8%, *P* < 0.001; Month 1: *Q* = 32.05; df = 3, *I*^2^ = 90.6%, *P* < 0.001). An examination of publication bias was carried out by visual observation of the funnel plot in which the standard error of the SMD of each study was plotted against its SMD (Fig. 2). Egger’s regression test and Begg’s test suggested that there was no significant bias (Egger: −2.397, 95% CI: −6.072 to −1.277; *t* = −1.37, *P* = 0.187; Begg: −1.56, *P* = 0.119). Two outliers were detected (Pathak *et al.*, 2021; Peters *et al.*, 2022). Differences in effect sizes might be due to variations in the medication’s administration method or the usage of different measurement instruments. We conducted sensitivity analyses after excluding these studies, and the pooled effect size remained significant (Cohen’s *d* = −0.94; 95% CI: −1.25 to −0.64; *P* < 0.001; *I*^2^ = 77.7%).

Risk of bias was evaluated for 13 double-blind RCTs, and 8 open-label studies; 14 received a good quality rating, and 7 received a fair quality grade (Table S3, Table S4). The absence of intention-to-treat in randomized individuals and the lack of sample-size justification in the trials were both frequent possible sources of bias in these investigations.

### Moderator effects

**Figure 3.**
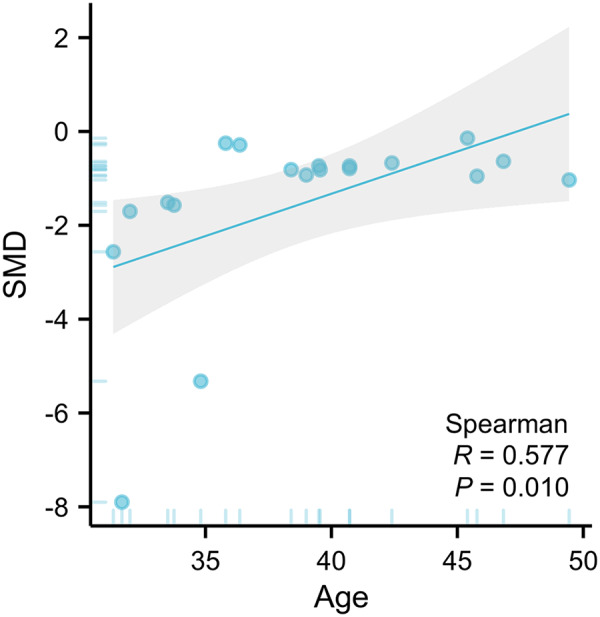
An illustration of the association between age of participants and SMD.

The primary moderator analysis confirmed that younger age significantly enhanced ketamine’s anti-suicidal efficacy (*β* = 0.158 per year, 95% CI: 0.01–0.31; *P* = 0.038, Fig. 3). The evaluation time points and the frequency of medication administered did not moderate the main treatment effect (Table 2; Figure S1).

**Table 2.** Moderator analyses

Moderator	β (95% CI)	SE	*P* value
Time points	−0.109 (−0.378, 0.160)	0.127	0.404
Age	0.158 (0.010, 0.306)	0.070	**0.038**
Frequency of medication	0.132 (−0.205, 0.468)	0.160	0.421

The bold values indicate statistical significance with p < 0.05.

### Secondary analyses

Results of the secondary analyses are summarized in Table 3. In brief, ketamine treatment showed greater benefits in participants with a high risk of suicide. Health status of participants, study design and control medication also moderated the overall effect (*P* < 0.05). Open-label trials reported nearly double the effect size of double-blind RCTs (Cohen’s *d* = − 1.461 vs. − 0.697; *P* < 0.001), suggesting unblinding may amplify placebo effects or clinician-reported improvements. Similarly, studies with ‘no control’ arms overestimated efficacy (Cohen’s *d* = −1.554) compared to placebo-controlled trials (Cohen’s *d* = −0.671; *P* < 0.001), further supporting potential bias in uncontrolled designs.

**Table 3.** Secondary analyses

			Effect size			Test(s) of heterogeneity	
Moderator group	Moderator variable	No. of studies	Cohen’s d	95% CI	*P*	*Q*	*P*
Health status	MDD	9	−0.766	−0.971, −0.561	**<0.001**	29.85	**<0.001**
	Active SI	3	−2.107	−2.720, −1.493	**<0.001**		
	TRD	4	−0.860	−1.162, −0.558	**<0.001**		
	Other	4	−1.410	−1.636, −1.184	**<0.001**		
Administration treatment	Single	12	−1.042	−1.230, −0.855	**<0.001**	0.14	0.718
	Repeated	8	−1.092	−1.279, −0.905	**<0.001**		
Assessment tool	Other	3	−1.293	−1.785, −0.802	**<0.001**	6.68	0.083
	BSI	13	−1.065	−1.214, −0.916	**<0.001**		
	CSSRS	2	−0.143	−0.913, 0.627	**<0.001**		
	MADRS-SI	2	−1.186	−1.595, −0.776	**<0.001**		
Study design	Double-blind RCTs	11	−0.697	−0.881, −0.512	**<0.001**	31.77	**<0.001**
	Open-label study	8	−1.461	−1.651, −1.271	**<0.001**		
Administration route	Intravenous	16	−1.138	−1.282, −0.995	**<0.001**	6.37	0.095
	Oral	1	−0.790	−1.623, 0.044	0.063		
	Intramuscular	1	−0.735	−1.564, 0.094	0.082		
	Intranasal	2	−0.621	−1.038, −0.204	**0.004**		
Control	Placebo	6	−0.671	−0.981, −0.361	**<0.001**	34.98	**<0.001**
	Midazolam	6	−0.774	−0.993, −0.555	**<0.001**		
	ECT	1	−0.762	−1.350, −0.175	**0.011**		
	None	6	−1.554	−1.763, −1.345	**<0.001**		

BSI, Beck Scale for Suicidal Ideation; CSSRS, Columbia-Suicide Severity Rating Scale; ECT, emission computed tomography; MADRS-SI, Montgomery-Asberg Depression Rating Scale – Suicidal Ideation; MDD, major depressive disorder; SI, suicide ideation; TRD, treatment-resistant depression. The bold values indicate statistical significance with p < 0.05.

Health status further stratified responses that patients with active SI exhibited the largest reductions (Cohen’s *d* = −2.107, 95% CI: −2.720 to −1.493), followed by those with TRD (Cohen’s *d* = −0.860) or major depressive disorder (MDD, Cohen’s *d* = −0.766).

### Adverse events

The reported adverse events (such as nausea, dizziness, headache, anxiety, etc.) were used to gauge the ketamine’s safety. Dissociation (38.8%, 95% CI: 7.8%–69.8%, *P* = 0.014, very low–quality evidence), nausea (31.6%, 95% CI: 20.4%–42.8%, *P* < 0.001, very low–quality evidence), dizziness (24.7%, 95% CI: 8.7%–40.8%, *P* = 0.003, low–quality evidence), headache (22.0%, 95% CI: 5.1%–39.0%, *P* = 0.011, very low–quality evidence) and anxiety (15.8%, 95% CI: 8.2%–23.5%, *P* < 0.001, very low–quality evidence) were the most often reported side effects. The proportion of reported adverse events are summarized in Table S6.

## Discussion

In this meta-analysis, we investigated the efficacy of ketamine treatment in reducing SI in high-risk populations. Our analysis of 21 studies showed a significant decrease in SI following ketamine treatment compared to placebo or other control conditions. However, the anti-suicide effects of ketamine were not sustained beyond a mean follow-up of 2 weeks. This finding contrasts with reports of longer-lasting antidepressant effects in other reviews (Li *et al.*, 2022; Lima *et al.*, 2022), though these conclusions derive from entirely distinct study cohorts (0/21 overlapping studies) focused on depression outcomes rather than SI. The certainty of evidence was downgraded to low due to substantial between-study heterogeneity (*I*^2^ = 89%) and methodological limitations (e.g., 38% open-label designs). While sensitivity analyses excluding outliers confirmed robustness (SMD = −0.94; *P* < 0.001), generalizability to real-world settings remains constrained by stringent trial protocols.

Although the therapeutic effect of ketamine is significant, the precise mechanisms by which ketamine exerts its anti-suicidal effects are not yet fully understood. Ketamine rapidly inhibits indoleamine 2,3-dioxygenase (IDO-1), a key enzyme in the kynurenine pathway, within 4 hours of administration. This suppression reduces the production of neurotoxic quinolinic acid (QUIN)– a potent N-methyl-D-aspartate (NMDA)receptor agonist linked to glutamate excitotoxicity and neuronal apoptosis – while increasing KYNA, an NMDA antagonist with neuroprotective properties (Brown *et al.*, 2024; Savitz, 2020). Recent studies demonstrate that this acute rebalancing of QUIN/KYNA ratios correlates with rapid SI reduction in high-risk populations, independent of depressive symptom trajectories (Brown *et al.*, 2024). Kynurenine pathway modulation also explains ketamine’s transient efficacy: QUIN/KYNA ratios normalize within 2 weeks, mirroring the observed decline in anti-suicidal effects.

Our moderator analyses suggest that ketamine treatment has a more significant impact on reducing SI in younger populations than older ones. This finding is consistent with an RCT study that found that the effect of ketamine on reducing SI decreased with rising age in men (Derntl *et al.*, 2019). One possible explanation for this age-related difference is that ketamine’s anti-suicide mechanism may involve modulating glutamatergic signalling, which may decline with age, leading to attenuated neuroplasticity pathways. This is supported by research showing that the integrity of signalling pathways in the hippocampus, such as those involving PPARα and F3/Contactin, is crucial for the action of antidepressants and can be compromised with age or stress (Chen *et al.*, 2022; Wang *et al.*, 2021). Therefore, age should be considered when designing treatment approaches for different age groups to optimize the effectiveness of ketamine therapy.

It is also worth noting that ketamine’s rapid onset of action may be particularly beneficial for individuals with active SI, as it can provide almost immediate relief from symptoms. This may be especially important for individuals at high risk of suicide, as they may not have the time to wait for traditional antidepressant medications to take effect.

Despite the promising results of this study, there are several limitations that should be acknowledged. The sample size of this meta-analysis was relatively small, which may limit the generalizability of the findings. In some comparisons, statistical heterogeneity was high. For this reason, a random-effects model was used to interpret the results of the comparisons with high heterogeneity, yet, future randomized studies with better designs will be required to further assess these outcomes. The majority of the studies included in this meta-analysis were conducted in a controlled setting with strict monitoring and supervision, which may not reflect real-world clinical practice. Finally, the long-term side effects of ketamine treatment for SI have not been fully evaluated, and further research is needed to address these issues. Our analysis revealed a notable gap in the assessment of long-term outcomes. Among the included studies, only one (Shivanekar *et al.*, 2022) reported follow-up data extending to 6 months, and no studies systematically investigated potential long-term consequences such as the development of dependence, tolerance, or urinary tract toxicity with repeated administration. While acute adverse events (e.g., dissociation, nausea) were well-documented, the current evidence base is insufficient to characterize the long-term safety profile of ketamine for SI.

## Conclusion

In summary, our meta-analysis offers support for the effectiveness of ketamine treatment in decreasing SI among high-risk individuals. Although further research is necessary to clarify the mechanisms underlying the effects of ketamine, the swift and powerful effects of this intervention on SI suggest that it holds considerable promise for high-risk patients. Nevertheless, clinicians must weigh the potential risks and long-term consequences of ketamine treatment carefully.

## Supporting information

10.1017/S2045796025100371.sm001 Tang et al. supplementary material Tang et al. supplementary material

## Data Availability

Extracted data are available on request to the corresponding author.

## Supplementary material

The supplementary material for this article can be found at https://doi.org/10.1017/S2045796025100371.
