# Supplementary material for: Ketamine treatment alleviates suicide ideation in high-risk populations: a systematic review and meta-analysis
Source: Epidemiol Psychiatr Sci. 2026 Jan 12;35:e6. doi: 10.1017/S2045796025100371 (PMC12816934; doi:10.1017/S2045796025100371)

# Supplementary appendix

Table of Contents

[Section 1. List of acronyms and abbreviations 2](#_Toc133436221)

[Section 2. Preferred Reporting Items for Systematic Reviews and Meta-Analyses (PRISMA) compliance 3](#_Toc133436222)

[Section 3. Supplementary figures and tables 7](#_Toc133436223)

## Section 1. List of acronyms and abbreviations

| **Abbreviation/acronym** | **Full phrase** |
| --- | --- |
| ASIQ | Adult Suicide Ideation Questionnaire |
| BSI | Beck Scale for Suicidal Ideation |
| CI | confidence interval |
| CSSRS | Columbia-Suicide Severity Rating Scale |
| HDRS-SI | Suicide item of the Hamilton Depression Rating Scale |
| IM | Intramuscular |
| IN | Intranasal |
| MADRS-SI | Suicide item of the Montgomery–Asberg Depression Rating Scale |
| MDD | Major depressive disorder |
| MSSI | Modified Scale for Suicidal Ideations |
| PTSD | post-traumatic stress disorder |
| QIDS-SI | Quick inventory of depressive symptoms suicidality item |
| RCT | randomized controlled trial |
| SD | standard deviation |
| SMD | standard mean difference |
| SPS | Suicide Probability Scale |
| SI | Suicide ideation |
| TRD | Treatment-resistant depression |
| World Health Organization | WHO |

## Section 2. Preferred Reporting Items for Systematic Reviews and Meta-Analyses (PRISMA) compliance

| **Section and Topic** | **Item #** | **Checklist item** | **Location where item is reported** |
| --- | --- | --- | --- |
| **TITLE** | | |  |
| Title | 1 | Identify the report as a systematic review. | Paper title |
| **ABSTRACT** | | |  |
| Abstract | 2 | See the PRISMA 2020 for Abstracts checklist. | N/A |
| **INTRODUCTION** | | |  |
| Rationale | 3 | Describe the rationale for the review in the context of existing knowledge. | Main text introduction, paragraph 2-3 |
| Objectives | 4 | Provide an explicit statement of the objective(s) or question(s) the review addresses. | Final sentence of main text introduction |
| **METHODS** | | |  |
| Eligibility criteria | 5 | Specify the inclusion and exclusion criteria for the review and how studies were grouped for the syntheses. | Main text methods  “Eligibility criteria” and “Exclusion criteria” section |
| Information sources | 6 | Specify all databases, registers, websites, organisations, reference lists and other sources searched or consulted to identify studies. Specify the date when each source was last searched or consulted. | Main text “Methods” paragraph 1 |
| Search strategy | 7 | Present the full search strategies for all databases, registers and websites, including any filters and limits used. | Supplementary appendix “Table S1 Search details” |
| Selection process | 8 | Specify the methods used to decide whether a study met the inclusion criteria of the review, including how many reviewers screened each record and each report retrieved, whether they worked independently, and if applicable, details of automation tools used in the process. | Main text methods “Search strategy” and “Study selection and data extraction” |
| Data collection process | 9 | Specify the methods used to collect data from reports, including how many reviewers collected data from each report, whether they worked independently, any processes for obtaining or confirming data from study investigators, and if applicable, details of automation tools used in the process. | Main text methods “Study selection and data extraction” |
| Data items | 10a | List and define all outcomes for which data were sought. Specify whether all results that were compatible with each outcome domain in each study were sought (e.g. for all measures, time points, analyses), and if not, the methods used to decide which results to collect. | Main text methods “Study selection and data extraction” |
|  | 10b | List and define all other variables for which data were sought (e.g. participant and intervention characteristics, funding sources). Describe any assumptions made about any missing or unclear information. | Main text methods “Study selection and data extraction” |
| Study risk of bias assessment | 11 | Specify the methods used to assess risk of bias in the included studies, including details of the tool(s) used, how many reviewers assessed each study and whether they worked independently, and if applicable, details of automation tools used in the process. | Main text “Assessment of bias and methodological quality” |
| Effect measures | 12 | Specify for each outcome the effect measure(s) (e.g. risk ratio, mean difference) used in the synthesis or presentation of results. | Main text “Study selection and data extraction” paragraph 1 |
| Synthesis methods | 13a | Describe the processes used to decide which studies were eligible for each synthesis (e.g. tabulating the study intervention characteristics and comparing against the planned groups for each synthesis (item #5)). | N/A |
|  | 13b | Describe any methods required to prepare the data for presentation or synthesis, such as handling of missing summary statistics, or data conversions. | Main text “Study selection and data extraction” paragraph 1 |
|  | 13c | Describe any methods used to tabulate or visually display results of individual studies and syntheses. | Main text methods “Data analysis” |
|  | 13d | Describe any methods used to synthesize results and provide a rationale for the choice(s). If meta-analysis was performed, describe the model(s), method(s) to identify the presence and extent of statistical heterogeneity, and software package(s) used. | Main text methods “Data analysis” |
|  | 13e | Describe any methods used to explore possible causes of heterogeneity among study results (e.g. subgroup analysis, meta-regression). | Main text methods “Data analysis” |
|  | 13f | Describe any sensitivity analyses conducted to assess robustness of the synthesized results. | Main text methods “Data analysis” |
| Reporting bias assessment | 14 | Describe any methods used to assess risk of bias due to missing results in a synthesis (arising from reporting biases). | Main text methods “Data analysis” |
| Certainty assessment | 15 | Describe any methods used to assess certainty (or confidence) in the body of evidence for an outcome. | Main text methods “Assessment of bias and methodological quality” |
| **RESULTS** | | |  |
| Study selection | 16a | Describe the results of the search and selection process, from the number of records identified in the search to the number of studies included in the review, ideally using a flow diagram. | Main text methods “Results” paragraph 1 |
|  | 16b | Cite studies that might appear to meet the inclusion criteria, but which were excluded, and explain why they were excluded. | N/A |
| Study characteristics | 17 | Cite each included study and present its characteristics. | Table 1 |
| Risk of bias in studies | 18 | Present assessments of risk of bias for each included study. | Supplementary appendix  Table S3-S4 |
| Results of individual studies | 19 | For all outcomes, present, for each study: (a) summary statistics for each group (where appropriate) and (b) an effect estimate and its precision (e.g. confidence/credible interval), ideally using structured tables or plots. | Figure 1 |
| Results of syntheses | 20a | For each synthesis, briefly summarise the characteristics and risk of bias among contributing studies. | Table 1, Main text results |
|  | 20b | Present results of all statistical syntheses conducted. If meta-analysis was done, present for each the summary estimate and its precision (e.g. confidence/credible interval) and measures of statistical heterogeneity. If comparing groups, describe the direction of the effect. | Table 1, Main text results |
|  | 20c | Present results of all investigations of possible causes of heterogeneity among study results. | NA |
|  | 20d | Present results of all sensitivity analyses conducted to assess the robustness of the synthesized results. | Main text results |
| Reporting biases | 21 | Present assessments of risk of bias due to missing results (arising from reporting biases) for each synthesis assessed. | NA |
| Certainty of evidence | 22 | Present assessments of certainty (or confidence) in the body of evidence for each outcome assessed. | 95% uncertainty intervals (UIs) are presented for all mean estimates in the main text results section and in all figures, as relevant |
| **DISCUSSION** | | |  |
| Discussion | 23a | Provide a general interpretation of the results in the context of other evidence. | Main text discussion, paragraph 2-4 |
|  | 23b | Discuss any limitations of the evidence included in the review. | Main text discussion, paragraph 5 |
|  | 23c | Discuss any limitations of the review processes used. | Main text discussion, paragraph 5 |
|  | 23d | Discuss implications of the results for practice, policy, and future research. | Main text conclusion |
| **OTHER INFORMATION** | | |  |
| Registration and protocol | 24a | Provide registration information for the review, including register name and registration number, or state that the review was not registered. | Main text Methods, paragraph 1 |
|  | 24b | Indicate where the review protocol can be accessed, or state that a protocol was not prepared. | Main text Methods |
|  | 24c | Describe and explain any amendments to information provided at registration or in the protocol. | N/A |
| Support | 25 | Describe sources of financial or non-financial support for the review, and the role of the funders or sponsors in the review. | Main text acknowledgments |
| Competing interests | 26 | Declare any competing interests of review authors. | Main text declaration of interest |
| Availability of data, code and other materials | 27 | Report which of the following are publicly available and where they can be found: template data collection forms; data extracted from included studies; data used for all analyses; analytic code; any other materials used in the review. | N/A |

## Section 3. Supplementary figures and tables

**Table S1 Search details**

| **Pubmed** | | |
| --- | --- | --- |
|  | **Search string** | **Result** |
| #1 | ((ketamine[Title/Abstract]) OR (esketamine[Title/Abstract])) OR (racemic ketamine[Title/Abstract]) | 26,070 |
| #2 | suicid*[Title/Abstract] | 111,675 |
| #3 | Review[Publication Type] | 3,729,893 |
| #4 | (#1 AND #2) NOT #3 | **494** |
| **Web of Science** | | |
| #1 | (TS=(ketamine)) OR TS=(esketamine) | 42,137 |
| #2 | ((TS=(suicide)) OR TS=(suicide ideation)) OR TS=(suicidal ideation) | 168,769 |
| #3 | DOP=(2003-04-01/2025-10-01) | 55,719,123 |
| #4 | #3 AND #2 AND #1 and Preprint Citation Index (Exclude – Database) and Research Commons (Exclude – Database) and Letter or Book or News or Meeting or Review Article (Exclude – Document Types) and Article (Document Types) | **564** |
| **EMBASE** | | |
| #1 | esketamine:ab,ti OR ketamine:ab,ti | 31,431 |
| #2 | suicide:ab,ti OR 'suicidal ideation':ab,ti | 100,590 |
| #3 | #1 and #2 | 543 |
| #4 | #3 AND 'human'/de | **514** |
| **medRxiv** | | |
|  | term "ketamine" and abstract or title "suicid" (match any words) and posted between "01 Apr, 2003 and 01 Oct, 2025" | **97** |

**Table S2. Definitions and codes for the moderators**

| **Primary moderators** | **Definition** |
| --- | --- |
| Time points | Time since the last dose |
| Age | Mean age of participants |
| Frequency of medication | The number of times the patient received medication over the course of the intervention |
| **Secondary moderators** | **Definition** |
| Health status |  |
| MDD | The participants had major depressive disorder |
| Active SI | The participants had active suicide ideation |
| TRD | The participants had treatment-resistant depression |
| Other | The participants present any other physical or mental condition or both |
| Administration treatment |  |
| Single | The patient was given a single dose |
| Repeated | The patient was given repeated doses |
| **Assessment tool** |  |
| BSI | Suicidal ideation was measured using the Beck Scale for Suicidal Ideation |
| CSSRS | Suicidal ideation was measured using the Columbia-Suicide Severity Rating Scale |
| MADRS-SI | Suicidal ideation was measured using the Suicide item of the Montgomery–Asberg Depression Rating Scale |
| Other | Suicidal ideation was measured using other Scales |
| **Study design** |  |
| Double-blind RCTs | The study was a double-blind randomized controlled trial |
| Open-label study | The study was open-label study |
| **Administration route** |  |
| Intravenous | Ketamine was administered intravenously |
| Oral | Ketamine was administered orally to the patient |
| Intramuscular | The patient was given ketamine by intramuscular injection |
| Intranasal | Ketamine was administered intranasally |
| **Control** |  |
| Placebo | Patients in the control group received normal saline as placebo. |
| Midazolam | Patients in the control group received midazolam |
| ECT | Patients in the control group received electroconvulsive therapy |
| None | The before-after (pre-post) studies with no control group |

**Table S3. Risk of bias assessment for controlled intervention studies**

| **Study** | **1** | **2** | **3** | **4** | **5** | **6** | **7** | **8** | **9** | **10** | **11** | **12** | **13** | **14** | **Rating** |
| --- | --- | --- | --- | --- | --- | --- | --- | --- | --- | --- | --- | --- | --- | --- | --- |
| Ahmed GK, 2023 | **Y** | **NR** | **Y** | **Y** | **Y** | **Y** | **Y** | **Y** | **NR** | **NR** | **Y** | **Y** | **Y** | **NR** | Fair |
| Hochschild A, 2022 | **Y** | **CD** | **NR** | **Y** | **Y** | **Y** | **NR** | **NR** | **NR** | **Y** | **Y** | **Y** | **Y** | **NR** | Fair |
| Feeney A, 2021 | **Y** | **Y** | **Y** | **Y** | **Y** | **Y** | **Y** | **Y** | **NR** | **Y** | **Y** | **Y** | **Y** | **CD** | Good |
| Keilp JG, 2021 | **Y** | **NR** | **NR** | **NR** | **Y** | **Y** | **Y** | **Y** | **Y** | **Y** | **Y** | **Y** | **Y** | **NR** | Fair |
| Pathak U, 2021 | **Y** | **Y** | **Y** | **Y** | **Y** | **Y** | **Y** | **Y** | **Y** | **Y** | **Y** | **Y** | **Y** | **NR** | Good |
| Kheirabadi D, 2020 | **Y** | **CD** | **CD** | **NR** | **Y** | **Y** | **Y** | **N** | **Y** | **Y** | **Y** | **Y** | **Y** | **NR** | Fair |
| Ionescu DF, 2019 | **Y** | **Y** | **Y** | **Y** | **Y** | **Y** | **Y** | **Y** | **Y** | **Y** | **Y** | **Y** | **Y** | **NR** | Good |
| Canuso CM, 2018 | **Y** | **Y** | **Y** | **Y** | **Y** | **Y** | **Y** | **Y** | **NR** | **Y** | **Y** | **Y** | **Y** | **CD** | Good |
| Sinyor M, 2018 | **Y** | **CD** | **Y** | **Y** | **Y** | **Y** | **Y** | **Y** | **NR** | **Y** | **Y** | **Y** | **Y** | **CD** | Good |
| Fan W, 2017 | **Y** | **NR** | **Y** | **Y** | **Y** | **Y** | **Y** | **Y** | **NR** | **Y** | **Y** | **Y** | **Y** | **CD** | Good |
| Hu YD, 2016 | **Y** | **Y** | **Y** | **Y** | **Y** | **Y** | **Y** | **Y** | **CD** | **Y** | **Y** | **Y** | **Y** | **CD** | Good |
| Murrough JW, 2015 | **Y** | **CD** | **Y** | **Y** | **Y** | **Y** | **N** | **Y** | **Y** | **Y** | **Y** | **Y** | **Y** | **NR** | Good |
| Price RB, 2014 | **Y** | **CD** | **Y** | **Y** | **Y** | **Y** | **Y** | **Y** | **NR** | **Y** | **Y** | **Y** | **Y** | **NR** | Good |

Y, Yes; N, No; CD, cannot determine; NA, not applicable; NR, not reported

1. Was the study described as randomized, a randomized trial, a randomized clinical trial, or an RCT?

2. Was the method of randomization adequate (i.e., use of randomly generated assignment)?

3. Was the treatment allocation concealed (so that assignments could not be predicted)?

4. Were study participants and providers blinded to treatment group assignment?

5. Were the people assessing the outcomes blinded to the participants' group assignments?

6. Were the groups similar at baseline on important characteristics that could affect outcomes (e.g., demographics, risk factors, co-morbid conditions)?

7. Was the overall drop-out rate from the study at endpoint 20% or lower of the number allocated to treatment?

8. Was the differential drop-out rate (between treatment groups) at endpoint 15 percentage points or lower?

9. Was there high adherence to the intervention protocols for each treatment group?

10. Were other interventions avoided or similar in the groups (e.g., similar background treatments)?

11. Were outcomes assessed using valid and reliable measures, implemented consistently across all study participants?

12. Did the authors report that the sample size was sufficiently large to be able to detect a difference in the main outcome between groups with at least 80% power?

13. Were outcomes reported or subgroups analyzed prespecified (i.e., identified before analyses were conducted)?

14. Were all randomized participants analyzed in the group to which they were originally assigned, i.e., did they use an intention-to-treat analysis?

**Table S4. Risk of bias assessment for before-after (pre-post) studies with no control group**

| **Study** | **1** | **2** | **3** | **4** | **5** | **6** | **7** | **8** | **9** | **10** | **11** | **12** | **Rating** |
| --- | --- | --- | --- | --- | --- | --- | --- | --- | --- | --- | --- | --- | --- |
| Gaither R, 2022 | **Y** | **Y** | **Y** | **CD** | **Y** | **Y** | **Y** | **NR** | **Y** | **Y** | **Y** | **NA** | Good |
| Peters EM, 2022 | **Y** | **Y** | **Y** | **CD** | **Y** | **NR** | **Y** | **NR** | **Y** | **Y** | **Y** | **NA** | Fair |
| Shivanekar S, 2022 | **Y** | **Y** | **Y** | **N** | **Y** | **CD** | **Y** | **NR** | **N** | **Y** | **Y** | **NA** | Fair |
| Zhou Y, 2022 | **Y** | **Y** | **Y** | **CD** | **Y** | **Y** | **Y** | **N** | **Y** | **Y** | **Y** | **NA** | Good |
| Phillips JL, 2020 | **Y** | **Y** | **Y** | **CD** | **Y** | **Y** | **Y** | **N** | **Y** | **Y** | **Y** | **NA** | Good |
| Zhou Y, 2020 | **Y** | **Y** | **Y** | **CD** | **Y** | **Y** | **Y** | **N** | **Y** | **Y** | **Y** | **NA** | Good |
| Kashani P, 2014 | **Y** | **Y** | **Y** | **CD** | **Y** | **Y** | **Y** | **NR** | **Y** | **Y** | **Y** | **NA** | Good |
| Thakurta RG, 2012 | **Y** | **Y** | **Y** | **CD** | **Y** | **CD** | **Y** | **NR** | **Y** | **Y** | **Y** | **NA** | Fair |

Y, Yes; N, No; CD, cannot determine; NA, not applicable; NR, not reported

1. Was the study question or objective clearly stated?

2. Were eligibility/selection criteria for the study population prespecified and clearly described?

3. Were the participants in the study representative of those who would be eligible for the test/service/intervention in the general or clinical population of interest?

4. Were all eligible participants that met the prespecified entry criteria enrolled?

5. Was the sample size sufficiently large to provide confidence in the findings?

6. Was the test/service/intervention clearly described and delivered consistently across the study population?

7. Were the outcome measures prespecified, clearly defined, valid, reliable, and assessed consistently across all study participants?

8. Were the people assessing the outcomes blinded to the participants' exposures/interventions?

9. Was the loss to follow-up after baseline 20% or less? Were those lost to follow-up accounted for in the analysis?

10. Did the statistical methods examine changes in outcome measures from before to after the intervention? Were statistical tests done that provided p values for the pre-to-post changes?

11. Were outcome measures of interest taken multiple times before the intervention and multiple times after the intervention (i.e., did they use an interrupted time-series design)?

12. If the intervention was conducted at a group level (e.g., a whole hospital, a community, etc.) did the statistical analysis take into account the use of individual-level data to determine effects at the group level?

**Table S5 Prevalence of adverse events in ketamine treatment.**

| **Adverse events** | **Study** | **Prevalence (95% CI)** | ***P* value** |
| --- | --- | --- | --- |
| Nausea | 3 | 0.316 (0.204, 0.428) | **<0.001** |
| Dizziness | 5 | 0.247 (0.087, 0.408) | **0.003** |
| Dissociation | 3 | 0.388 (0.078, 0.698) | **0.014** |
| Headache | 4 | 0.220 (0.051, 0.390) | **0.011** |
| Anxiety | 4 | 0.158 (0.082 ,0.235) | **<0.001** |
| Paresthesia | 2 | 0.111 (-0.011, 0.232) | 0.074 |
| Sedation | 2 | 0.134 (0.045, 0.222) | **0.003** |
| Euphoric mood | 2 | 0.095 (0.014 ,0.176) | **0.022** |
| Constipation | 2 | 0.151 (-0.010, 0.311) | 0.066 |
| Diarrhea | 2 | 0.124 (-0.048, 0.297) | 0.158 |
| Restlessness | 2 | 0.067 (-0.060, 0.193) | 0.301 |
| Dry mouth | 2 | 0.147 (0.013, 0.280) | **0.031** |

**Figure S1. Effect of different follow-up periods.**


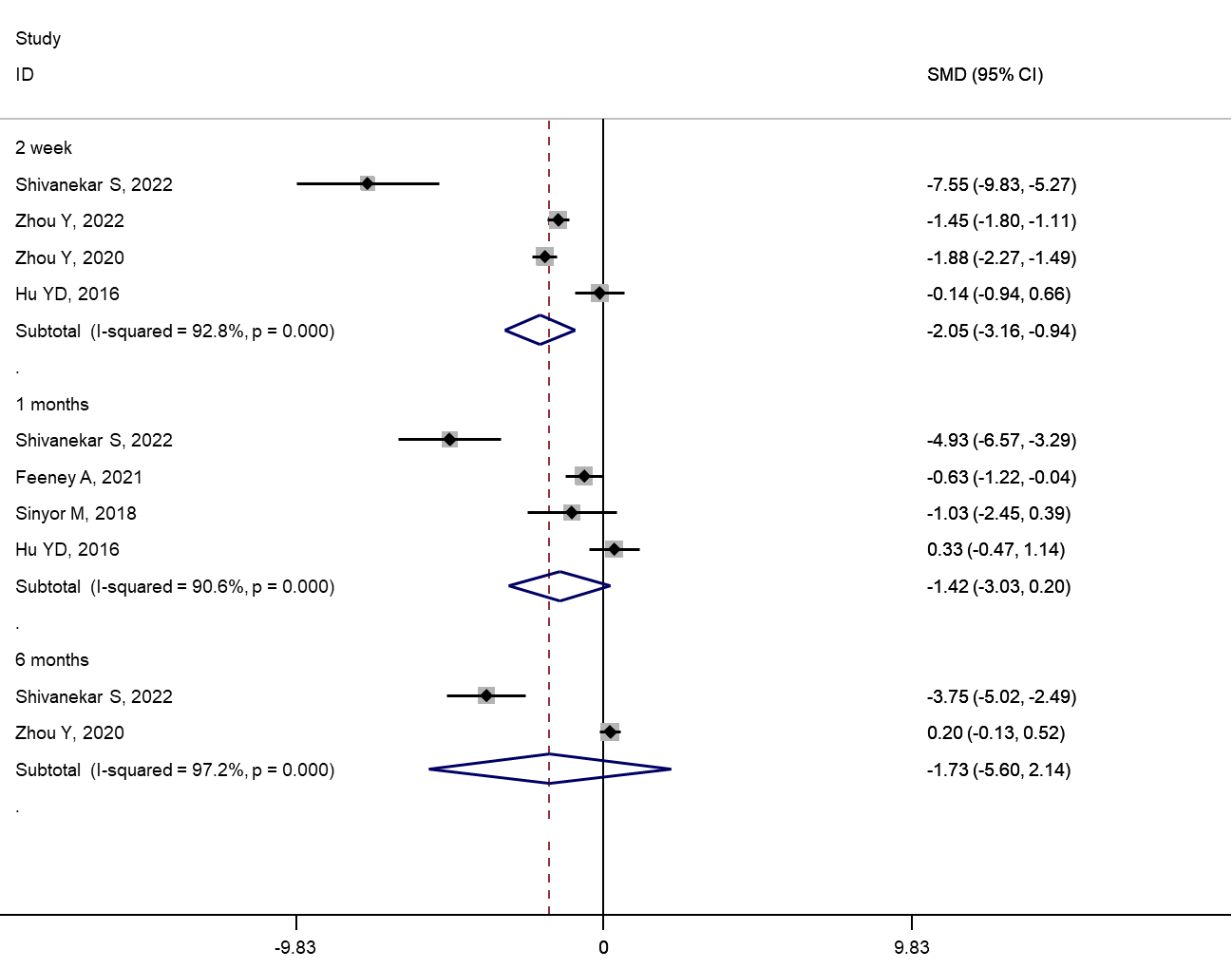

Supplement: Tang et al. supplementary material [file S2045796025100371sup001.docx]
